# Converging Human and Malaria Vector Diagnostics with Data Management towards an Integrated Holistic One Health Approach

**DOI:** 10.3390/ijerph15020259

**Published:** 2018-02-03

**Authors:** Konstantinos Mitsakakis, Sebastian Hin, Pie Müller, Nadja Wipf, Edward Thomsen, Michael Coleman, Roland Zengerle, John Vontas, Konstantinos Mavridis

**Affiliations:** 1 Hahn-Schickard, Georges-Koehler-Allee 103, 79110 Freiburg, Germany; Sebastian.Hin@Hahn-Schickard.de (S.H.); Roland.Zengerle@Hahn-Schickard.de (R.Z.); 2 Laboratory for MEMS Applications, IMTEK—Department of Microsystems Engineering, University of Freiburg, Georges-Koehler-Allee 103, 79110 Freiburg, Germany; 3 Department of Epidemiology and Public Health, Swiss Tropical and Public Health Institute, Socinstrasse 57, PO Box, 4002 Basel, Switzerland; pie.mueller@swisstph.ch (P.M.); nadja.wipf@swisstph.ch (N.W.); 4 University of Basel, Petersplatz 1, 4003 Basel, Switzerland; 5 Department of Vector Biology, Liverpool School of Tropical Medicine, Pembroke Place, Liverpool L3 5QA, UK; Edward.Thomsen@lstmed.ac.uk (E.T.); Michael.Coleman@lstmed.ac.uk (M.C.); 6 Institute of Molecular Biology and Biotechnology, Foundation for Research and Technology-Hellas, 70013 Heraklion, Greece; vontas@imbb.forth.gr (J.V.); mavridiskos@gmail.com (K.M.); 7 Pesticide Science Laboratory, Department of Crop Science, Agricultural University of Athens, 11855 Athens, Greece

**Keywords:** diagnostics (Dx), epidemics, information and communication technologies (ICT), insecticide resistance, malaria, One Health, vector-borne diseases

## Abstract

Monitoring malaria prevalence in humans, as well as vector populations, for the presence of *Plasmodium*, is an integral component of effective malaria control, and eventually, elimination. In the field of human diagnostics, a major challenge is the ability to define, precisely, the causative agent of fever, thereby differentiating among several candidate (also non-malaria) febrile diseases. This requires genetic-based pathogen identification and multiplexed analysis, which, in combination, are hardly provided by the current gold standard diagnostic tools. In the field of vectors, an essential component of control programs is the detection of *Plasmodium* species within its mosquito vectors, particularly in the salivary glands, where the infective sporozoites reside. In addition, the identification of species composition and insecticide resistance alleles within vector populations is a primary task in routine monitoring activities, aiming to support control efforts. In this context, the use of converging diagnostics is highly desirable for providing comprehensive information, including differential fever diagnosis in humans, and mosquito species composition, infection status, and resistance to insecticides of vectors. Nevertheless, the two fields of human diagnostics and vector control are rarely combined, both at the diagnostic and at the data management end, resulting in fragmented data and mis- or non-communication between various stakeholders. To this direction, molecular technologies, their integration in automated platforms, and the co-assessment of data from multiple diagnostic sources through information and communication technologies are possible pathways towards a unified human vector approach.

## 1. Introduction

Malaria is one of the most widespread infectious diseases, both in terms of geographical distribution and human cases. It is caused by a protozoan parasite of the species *Plasmodium (P.) falciparum*, *P. vivax*, *P. malariae*, and *P. ovale*. Among these, *P. falciparum* and *P. vivax* are the most prevalent, and *P. falciparum* the most pathogenic. *P. knowlesi* is a zoonotic malaria parasite that is known to spill over from its macaque reservoir host and may cause disease in humans. Malaria is a vector-borne disease transmitted from humans to humans through the bite of a female anopheline mosquito.

Despite the fact that malaria is a preventable and treatable disease, according to the World Health Organization (WHO) World Malaria Report 2017, there were 216 million cases and 445,000 death cases reported in 2016, with the African Region suffering approximately 90% of all malaria cases and deaths worldwide [1]. In addition to the human losses, malaria in endemic countries is a heavy burden to the healthcare systems, while it also inhibits socioeconomic development. The disease was considered as endemic in 91 countries in 2016, reduced from 108 in 2000. Much progress has been done during the past decade, with various malaria interventions leading to a 47% decline in malaria mortality rates globally between 2001 and 2013; however, the challenge still exists.

Although the major geographic distribution of malaria is assumed around the equator, it keeps being a latent global threat. Given the relation between malaria transmission and climate [2], the increase in global temperature is possibly causing tropical diseases and vectors to spread to higher altitudes in mountainous regions, and to higher latitudes that were previously spared. Furthermore, recent outbreaks of malaria in countries that had been malaria-free indicate the continuous threat of re-establishment of the disease in areas that were considered malaria-free [3]. The globalization and increased transportation of people are important risk factors for the disease spreading towards non-endemic regions [4,5]. Moreover, it is common in Europe and other high income countries to travel abroad for business or leisure, and that increases the probability of infections spreading due to returning travelers [6,7].

The challenges that prevent the rapid progress against malaria elimination are multi-factorial: (i) systemic: lack of sustainable investments and funding, poor functionality of healthcare systems, and unregulated environment are overall hurdles; (ii) biological: a major challenge presents with the ever-increasing resistance of parasites to antimalarial medicines, and of mosquitoes to insecticides; (iii) technical: lack of suitable tools to diagnose and treat efficiently, and lack of surveillance tools for evaluation of changes in prevalence, or gaps in the monitoring systems. A significant source of risk derives from patients infected with malaria parasites, but remain asymptomatic or undiagnosed. This group of people is unintentionally acting as an infectious parasite reservoir and contributing to the cycle of malaria transmission. Thus, for elimination, future diagnostic and treatment tools and strategies must undoubtedly be able to identify and aim at this target group as well, so as to clear malaria parasites from asymptomatic carriers. This will be strategically achieved through the adaptability of health systems and available tools, to prevent, detect, and treat not only the clinical cases, but every malaria infection, including the aforementioned asymptomatic ones.

Within this global landscape, the goals of the Global Technical Strategy for Malaria 2016–2030 [8] towards malaria elimination are built around three pillars: (1) ensure universal access to malaria prevention, diagnosis, and treatment; (2) accelerate efforts towards elimination and attainment of malaria-free status; (3) transform malaria surveillance into a core intervention for tracking of the disease and taking action in response to the data received. These pillars are supported by implementations for expansion of research and innovations towards key areas and strengthening the enabling environment, such as the health systems, capacity building, and empowerment of communities. 

Focusing on pillar 1 in our review, prevention includes vector control by means of insecticide-treated mosquito nets (ITNs) and long-lasting insecticide treated mosquito nets (LLIN), as well as indoor residual spraying (IRS). Diagnosis has to do with the timely, selective, and sensitive identification of the disease. Treatment refers to patient management, typically with artemisinin-based combination therapy (ACT), at the right dose, for efficient cure of the patient. 

Considering the malaria issue from an overall perspective, it is obvious that all three elements of pillar 1 have interdependencies. Population gets sick because vectors transmit the pathogens. Patients die because of lack of suitable diagnosis, which, in turn, leads to inappropriate treatment. This condition creates a complex landscape, involving humans and vectors. It cannot be denied that these are different aspects of the same issue. Observing the global research initiatives and funds, it is clear that there is only little done to combine the areas of human and mosquito diagnostics (Dx), even though both fields can benefit if treated in a converging manner. In fact, this type of thinking used to be the norm, with large scale field trials of integrated vector control and human interventions being supported in sub-Saharan Africa [9]. However, with the “failure” in Africa of the World Health Organization’s Global Malaria Elimination Plan [10], enthusiasm for holistic strategies waned, and is only now coming back in favor. One example is the study of Kramer et al., where the authors’ primary objective was, “*to evaluate the role of disease management (home-based management consisting of early detection and treatment), vector management (larviciding), and these two interventions in combination, in malaria control*” [11]. Very importantly, in the Global Technical Strategy for Malaria 2016–2030, the WHO recommends “*implementing two sets of interventions in a complementary way: (1) prevention strategies based on vector control, and, in certain settings and in some population groups, administration of chemoprevention, and (2) universal diagnosis and prompt effective treatment of malaria in public and private health facilities and at community level*” [8]. 

Focusing on the aforementioned recommendation by the WHO, this review aims to set some pathways towards the convergence of the fields of human malaria diagnostics and vector surveillance, and with easy adaptation and expansion to other vector-borne diseases. These pathways are as follows: (i) on the molecular diagnostics level, using suitable novel assays (and generally biochemical components) that are capable to provide multiplexed analysis for human and mosquito Dx; (ii) the integration of these assays and biochemical components into portable and automated platforms for deployment in the field; and (iii) the smart interfacing of acquired data through information and communication technologies (ICT) for strengthening the data management and surveillance interventions. 

We structure the content of this review according to these three pathways, and within each of them, we discuss commonly the human and vector side, in terms of current state-of-the-art and novel approaches, in order to demonstrate the close relation between these two fields. In Section 5, we provide some suggestions for the convergence through behavioral change and discuss the impact of the proposed holistic approach. The One Health, as a term, refers to “*the collaborative efforts of multiple disciplines working locally, nationally, and globally to attain optimal health for people, animals, plants and our environment*” [12,13]. As this special issue focuses on malaria, the authors directed their analysis towards this particular disease from the human and the vectors’ perspective as two branches of the One Health approach. Notably, the technologies that are reviewed and the interconnection that is suggested (i) between the technologies (vertically) and (ii) between human and vector diagnostics (horizontally) are applicable for other vector-borne diseases as well, therefore, the reader is urged to consider the impact and implications on the entire health system, rather than a single health facility/sentinel site level.

## 2. Diagnostics

### 2.1. State-of-the-Art and Challenges in Human Diagnostics

Symptomatic human *P. falciparum* infection may progress from a mild febrile illness to a severe and life-threatening disease. Therefore, it is recommended that malaria diagnosis should be available within a maximum of two hours after patients present themselves in the health facility [14]. Especially, because many patients might reach the health facility at a very late stage of the disease, rapid diagnosis and treatment are required. On the other hand, in highly endemic areas, a large proportion of the population can be infected, but yet have no symptoms, and therefore, a significant number of individuals may never be evaluated for infection, but remains as a reservoir of the parasites and indirect infection source to other people [8]. Assuming a correct diagnosis, the treatment of uncomplicated *P. falciparum* malaria consists of a quality-assured artemisinin-based combination therapy. However, increased resistance to antimalarial drugs (e.g., of *P. falciparum* to artemisinin in South-East Asia) [15] are threatening the efforts for malaria elimination, while rendering the treatment inefficient. In addition, the resistance patterns vary geographically, which makes the situation even more complicated, while there are currently no available tools for detecting resistances in combination with diagnosis.

Malaria diagnosis may become challenging in case there are co-infections or sudden outbreaks of epidemics, i.e., interference of other pathogens. This is why malaria should not be examined as an isolated event in diagnosis. In endemic countries, fever as symptom may be the result of several non-malaria infections. Some of them may exhibit local symptoms (e.g., gastrointestinal, respiratory, urinary tract infections), which facilitates differentiation from malaria infection. However these non-malaria febrile illnesses [16,17] are, in general, the most challenging ones to diagnose because of the so-called “fever of unknown origin”. Bacterial diseases, such as typhoid fever or pneumonia, often present the symptoms of malaria, and, in the absence of reliable diagnostic tests, the patient is given an antimalarial instead of an antibiotic treatment, which can have lethal consequences [18,19]. Moreover, typhoid fever (and salmonellosis in general) often occurs as malaria co-infection [20] and requires, therefore, to be identified even in the presence of a positive malaria test. In malaria-endemic regions, many patients are presumptively treated for malaria based on of febrile symptoms alone. Indicatively, the study of Reyburn et al. reports overdiagnosis of malaria in Tanzania, relating to the failure to treat alternative causes of severe infection [19]. During epidemics, a common procedure is to screen all patients for the epidemic-specific pathogen, but not for any other, thereby missing some co-infections, which may be proven lethal for the patient, as in some cases with dengue [21] and Ebola [22]. The consequence of misdiagnosis is wrong patient management (e.g., antimalarial when the patient actually suffers from viral infections; or antibacterial when the patient suffers from malaria). The impact of wrong treatment is the increase of morbidity and mortality [23], rise of antimicrobial resistance, and waste of precious pharmaceutical material [24]. 

The current gold standard in malaria diagnosis is microscopy blood smear tests [25]. In the African Region, the number of patients examined by microscopy increased from 33 million in 2010 to 48 million in 2014, while the global total was 203 million microscopy examinations of microscopy slides in 2014 [26]). Although this is one of the most “traditional” approaches, it requires experienced personnel and thorough sample preparation, while the method is specific only to detect malaria parasites. Furthermore, microscopy has poor sensitivity in low transmission settings and in asymptomatic patients, resulting in underestimation of disease prevalence. This is why tools with greater sensitivity than microscopy are needed in such cases, in order to detect these infection reservoirs [27]. Indicatively, the study of Okell et al. reports that for *P. falciparum*, microscopy underestimated prevalence by 50.8% compared to PCR [28].

Rapid diagnostic tests (RDTs) are lateral flow tests identifying the *Plasmodium*-specific antigens histidine-rich protein 2 (HRP2), lactate dehydrogenase (LDH), and aldolase. The matureness level of some malaria RDTs is quite high, and some of the tests are in the WHO recommendation list [29]. They are fast, cheap, easy-to-use, and mass producible, which justifies their broad use [30], and the reason why such tests will not be replaced by other techniques, especially for triage tests at remote sites (RDT manufacturers’ reports showed that RDT sales had more than doubled from 2011 to 2014 [30]). Yet, a major drawback of RDTs is that each test targets only one disease and, although typically the first RDT is given for malaria, in case of malaria-negative result, other RDTs have to be sought or other analyses to be done. Such procedures increase the overall cost as well as time-to-diagnosis and treatment, especially if the patient needs to travel to a more central health facility. In addition, and in cases of co-infections, malaria may be correctly diagnosed on one hand, but a co-existing viral or bacterial infection may be missed, thereby risking the follow-up treatment, if based only on a malaria-positive RDT. On top of these, and in the broad context of febrile illness diagnosis, we have to mention that apart from some of the malaria-specific RDTs, others, e.g., for chikungunya [31], are of disputed sensitivity or diagnostic value. This was found especially in asymptomatic patients, as indicated by the study of Cook et al., where the treatment of patients based in RDT diagnosis did not reduce the malaria incidence [32].

In case of bacterial infections, the gold standard is bacterial culture-based diagnosis. Though highly sensitive, since even a few cells will be eventually cultured, it is costly, requires much hands-on work and considerable user expertise, while the time-to-result may be up to 72 h. This method is only capable of detecting bacteria, thus, any co-infection with viruses or malaria will be missed.

The challenges identified in the above methods, presumed as gold standards, are that: (i) they all are time-consuming, (ii) they require experienced personnel to be operated, except for the RDTs; (iii) some of them (e.g., many RDTs) present sub-optimal sensitivity; and (iv) they all lack multiplexity, as they all detect only one specific disease per examination. Especially for the latter argument, in case of a malaria-negative result (as malaria is typically the first candidate disease tested in cases of febrile episodes), the health provider needs to consider follow-up sequential tests to identify the cause of fever, which adds time, cost, and risk of misdiagnosis and delayed (if at all) treatment. 

The solution to this major issue lies in the differential diagnosis of fever by means of (i) sensitive and specific identification of the pathogen (species) itself through detecting its genetic profile, and (ii) multiplexity, i.e., simultaneous detection of several potential fever-causing agents. These requirements are met by means of molecular diagnostic tests (Nucleic Acid Amplification Tests–NAAT [33,34,35]). Apart from their implementation in diagnosis, NAAT, in general, can be used in malaria programs and interventions for epidemiological research as well as surveys to map infections in low-transmission areas. Such methods could also be used for identifying foci for special interventions in elimination settings [36].

PCR started being employed in malaria diagnosis in the 2000s, and has proven to be very specific and sensitive, especially in cases of low parasitemia and co-infections. Even though microscopy and RDTs are still considered as the gold standards, PCR is often used as confirmatory method when results from traditional gold standard methods are unclear [37,38]. Currently in African laboratories there are two main PCR methods followed, namely the conventional PCR (gene amplified and detected using gel-electrophoresis, result is qualitative) and the real-time PCR (real-time monitoring of the reaction). The disadvantage of the former is that it takes place in an open system and requires tedious pre- and post-PCR sample handling and processing. This is overcome through real-time PCR using probes (e.g., FRET [39], TaqMan probe [40]), or intercalating dyes (e.g., SYBR green [41] real-time PCR), eliminating the need for post-PCR handling. Real-time PCR is currently the most widely used technique, not only for malaria detection and species identification and differentiation, but also for antimalarial drug resistance screening [42]. As it is carried out in closed environment (tubes) it is less prone to contamination and more suitable for automation. In addition, real-time PCR offers the possibility of high throughput and standardization [43], as well as quantification of the parasite load. This is of particular importance in the case of low-parasitemia patients, as well as when elimination (and not simply screening) is the scope of the test. An overview of malaria PCR can be found in the UNITAID 2016 Malaria Diagnostics Technology and Market Landscape Report [33]. 

Isothermal amplification technologies are comparatively novel methods that do not require thermocycling, like PCR, thus becoming more attractive in terms of reduced protocol complexity [44]. Due to their isothermal nature, they require lower power consumption than PCR, which renders them good candidates for resource- and infrastructure-limited settings. On the other hand, a main disadvantage of isothermal technologies is the fact that they cannot be quantitative, nor achieve a high degree of color-based multiplexing (e.g., 4-plex per reaction) like the real-time TaqMan probe PCR. Out of the available isothermal technologies currently being developed, the loop-mediated isothermal amplification (LAMP) [45,46] is the most frequently implemented in malaria diagnosis, mainly due to the fact that it uses 6 primer sets per target, thereby offering high specificity. It is compatible with turbimetry-based naked-eye detection, which requires a simplified detection instrumentation. The reaction takes place at a constant temperature of 64 °C. LAMP for malaria diagnosis has been used by various groups recently [47], in endemic areas such as India [48], Latin America [49], but even for detecting traveler-imported malaria in non-endemic areas, like Switzerland [50]. Recent studies report the development of real-time LAMP assays using SYBR green for real-time detection, rather than using visual color change, interestingly, amplifying the targets (*P. falciparum*) from directly boiled blood samples [51]. To this direction of real-time LAMP, one step has been further accomplished to detect other non-malaria fever-related tropical infections, such as dengue [52] and chikungunya [53], using reverse transcriptase real-time (RT)-LAMP.

### 2.2. State-of-the-Art and Challenges in Vector Diagnostics

Mosquito surveillance builds the foundation of every effective, efficient, and environmentally sustainable vector control program. Information on the species composition of the local vector population, alongside the knowledge on pathogen infection and insecticide resistance prevalence, is crucial for decision makers of vector control interventions [54]. 

Accurate species identification is essential to discriminate vector from non-vector species. Moreover, knowing the species composition can give an indication of the basic biology and behavior of a vector population. For example, breeding site preferences and host-seeking, biting, and resting behavior are often species-specific characteristics that offer potential targets for control interventions. Species identification is primarily carried out by microscopic examination of specimen using morphological keys. However, several African malaria vectors within the *An. gambiae* complex or *An. funestus* group present overlapping morphological characteristics and geographical distribution with other vectors and non-vectors. *Anopheles* spp. often represent a complex of several mosquito species that cannot be distinguished using morphological criteria. However, some of these species can occur sympatrically, and at the same time, they differ in their ability to transmit malaria and in their behavior (anthropophilic or zoophilic). For the task of species identification, the focus turned to molecular methods, with allele specific PCR (AS-PCR), restriction fragment length polymorphism PCR (RFLP-PCR) and multiplex TaqMan qPCR assays being the most used methods [54,55].

Regarding malaria, the detection at the infective stage of the four human-specific species *P. falciparum*, *P. vivax*, *P. ovale*, and *P. malariae* within *Anopheles* mosquito hosts worldwide (e.g., *An. gambiae*, *An. funestus*, *An. sacharovi*, *An. minimus*, *An. stephsensi*, *An. sinensis*, etc.) is of prime importance for vector control programs [54,55,56]. The detection of *Plasmodium*-infected mosquitoes has been traditionally performed by microscopically assessing the presence of sporozoites in dissected salivary glands [26]. This procedure may be infective stage-specific, but nowadays seems obsolete; as it is time- and resource-consuming, it requires experienced personnel and cannot differentially discriminate *Plasmodium* species. Enzyme-linked immunosorbent assays (ELISAs) targeting the infective stage specific circumsporozoite protein (CSP) of *P. falciparum* [57] or *P. vivax* [58] have been proposed as a rapid high-throughput method to screen potentially malaria-infected mosquitoes. However, these ELISAs have several limitations, as both their sensitivity and specificity are low, and most importantly, they have been shown to overestimate mosquito infection rates [59,60,61]. An alternative way to address the abovementioned limitations is the introduction of PCR-based molecular assays for the detection of *Plasmodium* in mosquitoes. Nested PCR approaches have been proposed [62], but they require two separate PCR reactions (low throughput, high cost), post-PCR interventions (agarose gel electrophoresis), and are prone to contamination, especially in a low-resources laboratory environment (no isolated/controlled PCR rooms). Single-round PCR approaches [63] have also been developed, but are less sensitive. Real-time PCR-based assays have been proposed as a more sensitive method of *Plasmodium* detection, with the TaqMan assay of Bass et al. being the most operationally relevant. This assay is rapid, requires no post-PCR processing, and can discriminate *P. falciparum* from *P. vivax*, *P. ovale*, and *P. malariae* [64]. However, because this method detects all mosquito stages of *Plasmodium*, the abdomen must be removed prior to extraction, in order to increase the chance of only detecting the infective sporozoites residing in the salivary glands e.g., in the head–thorax region. A novel TaqMan assay was recently developed, capable of detecting the infective stage of the *Plasmodium* parasite by measuring the mRNA levels that are specifically expressed at the infective parasite stage, the sporozoites. This method does not require head/thorax dissections, and can be used in mosquito pools, in single or multiplex formats, i.e., also targeting additional markers expressed in different tissues, such as detoxification enzymes associated with insecticide resistance [65].

Determination of insecticide resistance [66] using bioassays is a main component of Insecticide Resistance Management (IRM) programs. In general, bioassays can adequately determine if moderate to high levels of resistance are present, without investigating the underlying molecular mechanism. Insecticide resistance is usually assessed by two types of bioassays. Mosquitoes are either exposed to a single dose of an insecticide for a predefined time (prevalence bioassays) or mosquito mortality is recorded across a series of insecticide concentrations or exposure periods (quantifying bioassays). Prevalence bioassays are logistically easier to perform, but may be not as operationally informative as quantitative bioassays, which can be, in turn, impractical for widespread monitoring. In either case, bioassays involve methodological steps that are difficult to reproduce, require large numbers of live insects, can be affected from environmental factors, and cannot accurately detect low levels of resistance [67,68].

The underlying genetic mechanisms for insecticide resistance can be divided into two groups: target-site resistance and metabolic resistance. The sensitivity of insecticide target site is affected by non-silent point mutations, whereas the cause for metabolic resistance is overexpression of enzymes that detoxify or sequester insecticides. Molecular diagnostics for the determination of insecticide resistance can address most of these complexities, because they are more rapid, sensitive, and reproducible. They can detect resistance at its very early phase of development and monitor its increase. In this way, they can provide more reliable and evidence-based indications to whether changes are needed in insecticidal strategy, i.e., change of insecticidal regime due to detection of molecular alterations associated with the resistance to this particular insecticide. Again, multiplexing is essential for this task in order to determine the molecular aberrations that are causally related to insecticide resistance. Both target-site mutations and metabolic resistance can be robustly detected with the use of TaqMan-based molecular diagnostics [62,63,69]. 

A well-characterized mechanism of resistance to DDT and pyrethroids is caused by point mutations commonly referred to as knockdown resistance (kdr) mutations (L1014F, L1014S, N1575Y) of the insecticide target site, the para-type sodium channel. Detection of kdr mutations can be achieved by several assays that determine the presence of the mutant kdr alleles, such as the TaqMan assays, the AS-PCR, the heated oligonucleotide ligation assay (HOLA), the sequence specific oligonucleotide probe-enzyme-linked immunosorbent assay (SSOP-ELISA) and the PCR-dot blot [70,71,72,73].

Similarly, resistance to organophosphate and carbamate insecticides that can result from insensitivity of the target site enzyme, acetylcholinesterase (ace), can be detected by both TaqMan and conventional PCR-RFLP assays [74,75].

Determination of detoxification gene overexpression at the mRNA level can pinpoint aberrant expression of specific members of detoxification gene families in association with insecticide resistance. The main approaches that have been used to determine gene expression levels of genes associated with insecticide resistance are microarrays and singleplex qPCR using the SYBR Green chemistry, while ELISA immunodiagnostic protocols have been employed for the detection of detoxification enzymes in agricultural pests [76]. Microarrays have been used to screen insecticide resistant laboratory and field populations for detoxification gene deregulation; however, these do not represent a viable routine diagnostic approach, in terms of cost and complexity of analysis. Detoxification loci that have already been identified to be associated with insecticide resistance in several *Anopheles* species (e.g., the cytochrome P450 monooxygenases *CYP6P3*, *CYP6M2*, *CYP6P4*, and *CYP6Z*, and the glutathione-S transferase *GSTE2* in *An. gambiae*) may be targeted by a singleplex SYBR-Green based qPCR[77,78,79]. Multiplex TaqMan assays for the simultaneous determination of the mRNA expression levels of this “short list” of insecticide-resistant related genes are currently under development [80].

### 2.3. Progress beyond the State-of-the-Art

**Figure 1:**
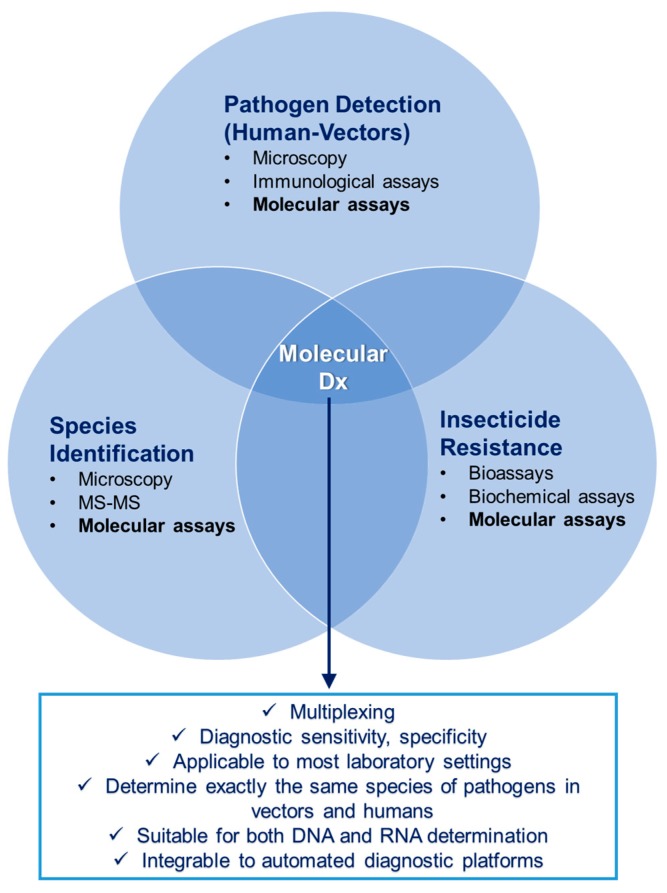
Molecular assays could represent a converging factor between human and vector diagnostics (common methodology for pathogen detection) and within vector diagnostics (common methodology for pathogen detection, vector species identification, insecticide resistance determination). “MS-MS” stands for tandem mass spectrometry. Source: K. Mavridis, FORTH.

Concluding from the aforementioned sections, the current needs and trends in both human and mosquito diagnostics, as one of the three proposed pathways to bridge these two fields, are (i) genetic profiling of pathogens (in case of human Dx) and species/resistance patterns (in case of mosquito Dx) by means of NAAT, and (ii) multiplexing of the NAAT. In order to establish a “diagnostic bridge”, the molecular assays seem ideal for several reasons (Figure 1):

1. They offer the capability of multiplexing, thus increasing throughput without the need of costly and complex analysis methodologies, such as microarrays.
2. They are diagnostically robust, they offer high sensitivity and specificity, they have been introduced in routine laboratory use for years, and are applicable to most laboratory settings.
3. The exact same assays that are used in human diagnostics for pathogen detection can also be used in vector diagnostics (exact same species/serotypes/lineages of pathogens can be detected), thus making epidemiological information more universal.
4. Both DNA and RNA determinations can be performed with exactly the same methodology from sample preparation to measurement.
5. Especially TaqMan PCR allows (semi)quantitative information about the degree of infection in humans, enabling a correlation between the Ct value and the pathogen concentration (parasite/bacterium/virus).
6. They can be integrated in an automated platform.

## 3. Packaging into Portable and Automated Platforms

### 3.1. Automation and Integration

The NAAT described in Section 2 are established on benchtop equipment and performed by experienced personnel, who use manual protocols mainly at central laboratories. In developed countries, this model is compatible with the degree of training of personnel, matureness of health systems, laboratory infrastructure, and affordability by the patients. However, in developing countries, the conditions are mostly prohibiting for use of such NAAT in central laboratories, because of the following reasons: (i) the personnel has often undergone limited training, therefore, the diagnostic procedure must be as easy-to-handle and as automated as possible; (ii) much of the population lives far from the capitals or big urban centers, therefore, the diagnosis must be done at the point-of-care (POC) instead of central laboratories; (iii) infrastructure may often be poor or not sustainably available (e.g., intermittent electricity), thus, the diagnostic process must be power-independent, or at least operable with alternative means of power, like solar energy; (iv) the environmental conditions (high temperature and humidity especially around the tropics) and lack of reagent storage facilities require independence from cold chain transport; (v) the per capita income of the population does not allow expensive diagnostic tests, thus, the affordability and production capacity must be taken care of. 

**Figure 2:**
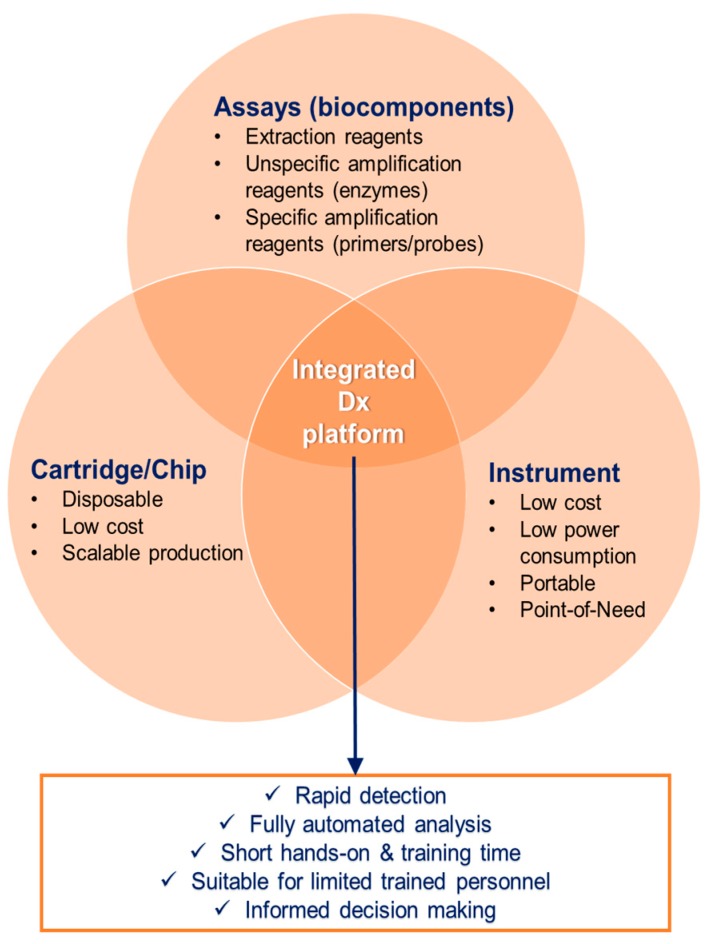
The “triangle” assays–cartridge–instrument lies in the heart of automated point-of-need platforms (here point-of-“need” is used as a more general term to include the mosquito diagnostics instead of point-of-“care” that is typically used for human diagnostics). This structure is common for any platform-oriented approach, and the concept of unified human and mosquito diagnostics can build upon this configuration. Source: K. Mitsakakis, Hahn-Schickard.

The above conditions have shaped the so-called ASSURED criteria by the WHO [81], which summarize some basic features that should be fulfilled by the “ideal” diagnostic test for the developing world, namely affordable by the target groups; sensitive and specific; user-friendly for limited trained personnel; rapid and robust; equipment-free; and delivered to those in need. For specific applications, there are specific Target Product Profiles (TPPs) composed by experts to describe the minimum requirements of diagnostic tools [82,83]. These requirements have led to the packaging of the aforementioned NAAT in integrated and automated platforms under the “triangle”: assays–cartridge–instrument (Figure 2). The “assays” perspective was described in Section 2. The “cartridge” and “instrument” are described in this section.

In terms of cartridges, most technologies are based on plastic or silicon disposable microfluidic chips [84]. Their fabrication technologies are usually rapid prototyping (for plastic), or standard MEMS (MicroElectroMechanical Systems) technologies (for silicon), which are then transferred to batch scale-up molding production. Such microfluidic cartridges are called micro total analysis systems (µTAS), and they integrate and miniaturize operations that would otherwise need an entire laboratory to be performed. In order to achieve this, assay developers and microsystems engineers collaborate in order to define the assay protocol, and break it down into sequential steps, which are then translated into so-called microfluidic unit operations [85]. The latter are interfaced towards the final sample-to-answer cartridge, which integrates the complete workflow required for NAAT, including sample preparation. The heterogeneous integration of all (bio)chemical components into the cartridge is a challenging, but necessary procedure, in order to achieve a fully integrated and automated analysis and avoid any user hands-on steps and interference. In particular, for molecular diagnostics integration, primers/probes are typically dry-stored [86], while unspecific amplification reagents, such as polymerase and reverse transcriptase enzymes, are typically stored in a lyophilized format [87,88]. In addition, for sample preparation using boom chemistry [89], nucleic acid extraction reagents need to be pre-stored as well. These are buffers for lysis of pathogens and subsequent purification of the DNA/RNA, as well as (magnetic) silica beads in case of bead-based extraction kits [90]. The integration of all biochemical processes imposes also the use of suitable controls, in order to ensure the non-failure of all the integrated procedures. These controls are typically in the form of extraction control, where a model organism undergoes the same extraction procedure as the unknown pathogens in the sample, and amplification control, where primers specific to this organism are amplified together (in the same amplification location/chamber) with the target [91,92].

The handling of cartridges is done by a dedicated instrument, which facilitates the temperature protocols for thermocycling, or isothermal conditions. The instrument also provides the environment for handling the microfluidics of the cartridge (capillary-, pressure-, or centrifugally-driven). Finally, it also integrates the modules for the multiplex real-time signal detection of nucleic acid amplification. Instruments utilizing infrared heating or contact heating can achieve rapid heating/cooling rates, thereby speeding up the real-time PCR. From the haptics perspective, the instrument engineering provides compact solutions, as some of the marketed near-patient or point-of-care devices are already portable or semi-portable (Table 1). Overall, the role of automated sample-to-answer platforms is to

1. Provide the suitable interface for safe and contamination-free sample inlet. The sample inlet should be of universal nature in order to facilitate analysis from different matrices, such as whole blood, serum, sputum (in case of tuberculosis), or swab transport medium (in case of respiratory infections), but also mosquito homogenate samples.
2. Automate sample preparation and interface it on the same cartridge with the NAAT mentioned in Section 2.
3. Integrate and *in situ* store (bio)chemical reagents that are required for sample-to-answer analysis.
4. Use a processing instrument, which incorporates the detection technology and reporting interface.
5. Be adaptable in exchanging the desired targets according to endemic and epidemic needs, and geographical requirements.

### 3.2. State-of-the-Art in Automated Detection Technologies

Screening the technological landscape, one can find several technologies for rapid analysis of nucleic acids. However, going deeper into the screening, the number of candidate technologies that can sufficiently respond to the requirements set out in Section 3.1 decreases significantly. Table 1 summarizes some of the main technologies and their performance characteristics. Inclusion criteria were that these specific technologies: (i) make use of NAAT; (ii) analyze at least in 2-plex level; (iii) are integrated sample-to-answer platforms, following the concept of Figure 2; (iv) are at late development, near-market, or at-market stage. From the listed technologies, only few are focusing on malaria and/or generally tropical infections for human diagnostics, and only one focuses on both human and vector diagnostics; that is why we choose to elaborate only on these in the current review. Mitsakakis et al. are preparing a more comprehensive overview of all these technologies [93]. For malaria diagnostic technologies at lower readiness levels, an overview is given in the 2016 UNITAID Malaria Diagnostics Technology and Market Landscape Report [33]. 

#### 3.2.1. VerePLEX^TM^ Biosystem

The platform has been developed through a joint effort of the STMicroelectronics, Italy, the Agency for Science, Technology and Research (A*STAR), Singapore, and Veredus Laboratories, Singapore. Several cartridges have been developed according to applications in demand (e.g., influenza, veterinary, biothreat agents). Among them, the VereTrop^TM^ chip includes a panel of 13 tropical diseases [94]. It is one of the very few platforms offering such degree of multiplexity for this kind of application. Technology-wise, the system needs two processing devices, one for the multiplex PCR and hybridization, and one for the CCD (Charged-Coupled Device) imaging-based microarray detection. This rises the total analysis time to approximately 3.5 h. The technology is currently at market stage.

#### 3.2.2. Q-POC^TM^

Q-POC™ from QuantumDx [95] is a handheld, battery-powered molecular diagnostic device, performing sample-to-answer DNA detection, offering, additionally, the option for drug susceptibility test. Its time-to-result is approximately 20 min, and its throughput analysis is based on its nanowire array biosensor chip. The nanowires are functionalized with nucleic acid probes to capture targeted sequences of genetic material, thereby enabling SNP and pathogen detection. The technology is capable of providing qualitative results for five malaria-causing species per disposable cartridge. QuantumDx aims to use the system at the primary care and for triage testing at the community level, where the majority of malaria patients are located. The technology is currently at near-market stage. 

#### 3.2.3. LabDisk

This centrifugal microfluidic platform has been/is being used in both fields of human and vector diagnostics. The LabDisk platform is a disk-shaped microfluidic cartridge that is handled by a LabDisk processing device, and offers a high degree of multiplexing based on geometric compartmentalization of the assays in distinct reaction chambers, in addition to color multiplexing per reaction chamber [96,97]. A recent version of the system makes use of 12 chambers (i.e., detection of up to 12 different human pathogens) which, in addition to 3-plex color multiplexing can give, in future, rise to a 36-plex detection configuration, especially for species identification, pathogen detection, and insecticide resistances. It has been recently used in some validation studies in Senegal and Sudan using a multiplex tropical infection panel (including malaria *P. falciparum*, *P. vivax*, *P. malariae* [98]), and is under development for the first proof of principle using mosquito samples [80]. The total time-to-answer is 2 h using LAMP. The technology is currently at late development stage.

## 4. Inclusion of Information and Communication Technologies

### 4.1. Clinical Algorithms for Patient Management

In a world of increasing connectivity, the information and communication technologies (ICT) play a significant role in the health sector. Considering that the results deriving from automated platforms like those described in Section 3 are simply numbers, there is a gap between these results and the clinical outcome. The ICT for health (otherwise termed “eHealth”, or “digital health”) can bridge this gap by means of suitable reporting tools, which take the diagnostic output data and combine/process them with suitable clinical algorithms in order to provide a decision support. Importantly, the aim of ICT is not to replace clinicians, but to assist them in decision making for patient management and rational prescriptions, especially in areas like malaria diagnosis in developing countries, where (i) the training of the clinicians may be limited; (ii) the possible causes of fever may not be malaria, or only malaria, but some other (co)-infections; (iii) epidemics often occur, whose pathogens exhibit the same symptoms like with malaria, thereby making clinical decisions inherently complicated. Such a decision support ICT platform is absolutely important, especially when data from multiplexed analyses need to be co-assessed, or when protein and nucleic acid analysis results need to be combined, along with the clinical symptoms and the history of the patient. Such platforms are operable on mobile systems, e.g., phones or, better, tablets, especially as the ICT penetration in developing countries is growing rapidly [99,100].

**Figure 3:**
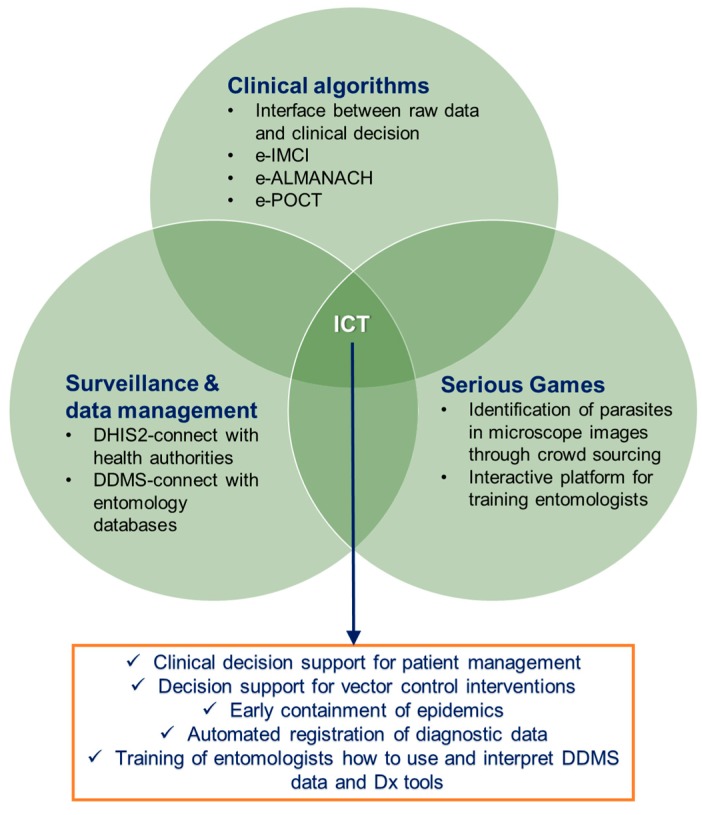
Aspects of information and communication technologies (ICT) related to the fields of human diagnostics and vector control. Source: K. Mitsakakis, Hahn-Schickard.

A detailed description of eHealth tools that have been/are being used for the management of fever is available at Mitsakakis et al. [93]. Some examples of such algorithms are as follows (Figure 3): The e-IMCI (“Integrated Management of Childhood Illness”), the computerized version of the IMCI developed by the WHO and UNICEF [101,102]. That platform did not take into account non-malaria causes of fever, such as the typhoid fever. Furthermore, viral diseases were not included in the e-IMCI, even though among children, the viral infections are one of the most common type of infections. Thus, the next generation of algorithm, the e-ALMANACH (ALgorithm for the MANAgement of CHildhood illness), was developed by Valérie D’Acremont and Blaise Genton at the Swiss Tropical and Public Health Institute [103]. The main upgrade was the inclusion of a broader set of possible diseases in the algorithm branch, based on the causes of global childhood mortality and morbidity from the Child Health Epidemiology Reference Group (CHERG). The impact of e-ALMANACH, in terms of health outcome and rational use of antimicrobials in the routine practice, was demonstrated through the drastic reduction of antibiotic prescriptions compared to the routine care [104]. An upgraded version of e-ALMANACH, called the e-POCT [105] combines (i) key clinical elements (demographics, symptoms and signs); (ii) input from sensors connected directly to a tablet and detecting parameters of severe illness (e.g., hypoxemia, tachycardia, severe anemia, and hypoglycemia); (iii) clinical predictor factors (e.g., fast breathing); (iv) results from a series of POC tests for host biomarkers, in particular, C-reactive protein (CRP) and procalcitonin (PCT). All these are used as data input into a tablet hosting the e-POCT algorithm. Its output provides (i) diagnostic classification(s) of disease(s) with its (their) probability; (ii) recommended immediate and/or hospital/home treatments (with dosage based on age/weight); and (iii) advice on admission versus discharge home. The algorithm is built into an android tablet using an Open Data Kit (ODK)-based software.

### 4.2. Surveillance and Data Management

#### 4.2.1. Surveillance in Human Diagnostics

Main features of epidemics are their sudden outbreak and rapid spread, therefore, close surveillance is mandatory, i.e., continuous, systematic collection, analysis, and interpretation of health data, confirmation and response to the epidemic, as well as planning, implementation, and evaluation of public health practices at any level of the health system. One way of epidemic surveillance is the early identification at the sentinel sites, which are health facilities or reporting sites, representative of an area, designated for early warning of pandemic/epidemic events. There, several epidemic-relevant pathogens (even those that cannot receive treatment, like dengue or Ebola viruses) are to be screened on a regular basis, and results be registered on a central server system. Over the long term, this would give valuable information to the authorities regarding potential outbreaks (e.g., if dengue levels in a community increase dramatically and unexpectedly within a short period of time). However, problems in the implementation were identified due to the low quality and integrity of data inserted in the platforms, being related to the manual handling of data deriving for example from RDTs. Towards this direction, and in order to strengthen the availability and use of surveillance and diagnostic data for identifying and reporting major diseases globally, the WHO Africa Regional Office set up the Integrated Disease Surveillance and Response (IDSR) [106], which started to be implemented in Africa in 2005 through an electronic surveillance tool called DHIS2 [107]. The DHIS2 (Figure 3) allows data management through visual elements such as charts, tables, and even maps, corresponding to geographical features. It enables the user to enter data from a variety of devices, thereby offering the opportunity to POC tests to be deployed at sentinel sites, and remotely send their acquired data in real-time to central data repositories. The latter are connected with the alarm units of health authorities, which will then be able to trace, early enough, the outbreak of an epidemic, and trigger actions in order to prevent it from expanding into a pandemic. The DHIS2, as well as several custom-built geographic information system (GIS)-based platforms, underpin the malaria surveillance systems in several countries (for review, see Ohrt et al. [108]). However, one inherent flaw in these systems is their focus only on disease in human populations. There is limited flexibility to incorporate entomological or intervention monitoring data, which is needed to support operational decision making.

#### 4.2.2. Data Management Systems in Entomology and Modern Communication Tools

Surveillance systems for insect vectors lag behind the digital technologies available for human disease surveillance. There are several examples of sophisticated vector surveillance tools that provide real-time decision support for vector control operations, but they typically exist in more developed countries. One example of such a system is the Gateway, the digital technology which underpins the California Vectorborne Disease Surveillance System in the United States (www.calsurv.org). This software has been in use by local vector control programs since 2006, and has been the foundation of operational decision-making. Decision support is facilitated by the rapid transformation of raw data into actionable information through the production of reports, graphs, and maps [109]. Unfortunately, in many malaria-endemic areas, the capacity to build and maintain such a system for the purposes of anopheline surveillance activities is lacking. As a result, a major problem has been the poor organization and integration of the large volumes of entomological data that are obtained in resource-limited malaria endemic countries every day, as well as the interpretation, utilization, and communication of these data. Often, different entomologists in a country store data in combinations of hard copies, or Microsoft Excel^®^ and Microsoft Access^®^ databases, resulting in a fragmented series of datasets. These data storage methods are unable to supply programs with accessible, consistent, or accurate data. Over time, a number of programs have gone on to develop fragmented systems to deal with different data sets as the program itself grows. This has led to an inherent problem of fragmented data, for which there is no easy way to reconcile and analyze in a systematic manner.

#### 4.2.3. An Integrated Data Management and Decision-Support System

The Disease Data Management System (DDMS) [110] is a data management software program (smart database) with the capability to collect data from routine entomological, epidemiological, and intervention monitoring activities, and store and make available stratified information on malaria risk and vector populations based on “user queries” in a standardized way [111]. It was designed to be as flexible as possible, and implementable with little existing technological capacity or infrastructure. It has been used by disease control programs to efficiently manage and integrate some entomological data and convert information into user-friendly formats for operational use [112]. The data can be presented to the users based in their “choice” (user queries) into customized reports, maps, graphs, images, and tables, providing, thus, a versatile and comprehensive picture of entomological and operational data to support operational decisions of disease control program members and stakeholders [80]. The DDMS database (Figure 3) can manage data at both the aggregated and individual level, including from other databases related to Public Health (such as Health Management Information Systems and Indoor Residual Spray Databases), thus avoiding duplication of effort and the implementation of parallel systems. 

Some of the features of the DDMS are (i) software flexibility, which allows full customization to local conditions; (ii) easy import of archived data from legacy systems; (iii) conversion of information into a wide range of reports, maps, and graphs; (iv) customized queries and reports; (v) full export function, which enables data to be used in other applications; (vi) strong geographic information system (GIS) component, which allows users to examine their data via an interactive map [80]. The impact of having a DDMS connected with systems of generating diagnostic and entomological data, in combination, is the continuous surveillance, monitoring, and evaluation using real-time data, the decision support for vector control strategies by malaria control programs, and the identification of disease prevalence, in order to plan and monitor specific interventions.

### 4.3. Serious Games

Effective vector control is dependent on insecticide resistance management (IRM), but in order to manage resistance, the core principles, appropriate methodologies, and benefits need to be well understood. Traditional communication and learning tools, such as guideline documents and large-scale workshops, have not accomplished the outcomes in IRM, which would be expected from such extensive education programs. The biological complexity of the molecular entomological data and insecticide resistance also present an obstacle to uptake within operational programs. The global community understands that the development of innovative methods of communication and learning to bridge this translational gap are urgently required. However, there is no current global consensus on how to individually or collectively address this.

The use of “Serious Games” (Figure 3) for improving communication and interpretation of data in public health initiatives is a growing trend [113]. According to Abt, 1970, “*Serious Games have an explicit and carefully thought-out educational purpose and are not intended to be played primarily for amusement. This does not mean that serious games are not, or should not be, entertaining*” [114]. Until 2014, Serious Games had never been introduced as innovative communication and educational tools to improve vector control programs. Nevertheless, the development of a Serious Game to assist African partner countries to better understand the use of data and to promote informed decisions for insecticide resistance management has been recently initiated by the Liverpool School of Tropical Medicine (LSTM), in cooperation with the Innovative Vector Control Consortium (IVCC). This Serious Game employs interactive ways of communicating guidelines and exemplifying good practices of successful insecticide resistance management use in the health sector, and has gone through initial development and successful pilot application testing in two resource-limited locations. The Game effectively communicates vector disease data and guidelines to national malaria control programs. It can be interfaced with disease data management systems and diagnostic tools and be used to educate end-user communities about the usefulness and value of these data, as well as teach the end-users how to best understand these data in terms of vector control and insecticide resistance management practices. Within the context of vector control, this Serious Game can assist traditional methods of decision making by: providing an interactive way of communicating and learning from guidelines and good practices for operational program levels; bridging translational gaps; and turning high level policy into effective operational practice. It also allows end-users to explore complex problems in a safe environment, allowing them to make mistakes and learn from them without real-world consequences [80].

Serious Games have also been implemented in the field of malaria detection in human diagnosis, for example, for parasite quantification, through an online game for analyzing images of infected thick blood smears. There, it was shown that, within 1 month, anonymous players from 95 countries played more than 12,000 games and generated a database of more than 270,000 clicks on the test images. Results revealed that combining 22 games from non-expert players achieved a parasite counting accuracy higher than 99% [115]. Along the same line, Mavandadi et al. developed a crowd-sourcing and distributed gaming platform that allows individuals from anywhere in the world to assist in identifying malaria infected red blood cells (RBCs) imaged under light microscopes [116]. 

## 5. Behavioral Change and Impact of a Holistic Approach

Even though there is evidence that the areas of human and mosquito diagnostics should not be treated separately, but there are several sub-areas that could be treated convergingly, such changes in mentality and strategy do not always take place easily and rapidly. For this reason, a set of behavioral changes should be accomplished mainly by the involved stakeholders. Some propositions are described below.

From the malaria control programs and NGOs perspective: Very positively, the WHO in its recently published Global Technical Strategy for Malaria 2016–2030 Agenda declares in pillar 1 that prevention strategies based on vector control and universal diagnosis are two sets of interventions that should be implemented in a complementary way [8]. More initiatives need to be taken, however, at operational levels closer to the health technologies. For example, NGOs from the human and vector diagnostics could cooperate and set up Target Product Profiles (TPPs), i.e., a set of performance specifications addressed to technology developers/manufacturers, for tools, assays, and ICT platforms that would be developed or upgraded along the direction of human/vector Dx convergence, so as to “synchronize” studies, analyses, data, and outcomes in these two sectors. In parallel, and to avoid reluctance from commercial partners/manufacturers, initiatives could be taken for updated business models that would take into account that commercial opportunities are enhanced when one considers both sectors as target groups for their products. This would give additional incentives to innovators to target both applications with the same intensity, and not underestimate one of the two.

From the intellectual property (IP) perspective, which is a key factor in innovation: The complicated IP situation with respect to integrated µTAS represents a major inhibiting factor for their introduction into low- and middle-income countries. When looking into the background IP of a mature sample-to-answer NAAT, a multitude of patents protect the integrated components. It is clear that stakeholders generating IP should be reimbursed for their efforts and ideas. However, it is, in general, difficult to find satisfying license agreements for all involved parties. An idea to solve this dilemma was proposed in the past by the Médécins Sans Frontières (MSF) with regard to so-called “fixed-dose combinations” (FDCs) [117] for effective treatment of HIV or tuberculosis. Patent holders may put their patents into such a pool so that such pills combine multiple drugs initially developed by different institutions or companies. Extrapolating from this example of pharmaceutical industries, lessons can be learned, and diagnostic developers/innovators, as well as assay developers from the human and the vector fields, may pool their IP in a similar manner and use the combined package of patents to develop, in this case, the integrated systems. Then, patent holders are reimbursed via license fees. There is a huge need to offer, in future, patent pools that collect the underlying patents and help to make them accessible for such systems. 

From the technology developer/innovator perspective: They should design their technologies in such a way as to be compatible with the diagnostic needs of both human and mosquito targets. For example, when designing malaria assays, amplification reagents should be as universal as possible. Similarly, the extraction kits for purifying the DNA/RNA should be made compatible with whole blood/serum (typical sample matrix for human malaria diagnosis) as well as mosquitoes freshly homogenized or after storage in RNA*later*^®^ (Ambion™ Inc., Austin, TX, USA)—an aqueous, non-toxic tissue storage reagent that rapidly permeates tissue to stabilize and protect RNA in unfrozen specimens—which is the typical sample matrix for mosquito analyses. The cartridges that process the human and the mosquito samples should be made as universal as possible, in terms of design and unit operations. This way, at the transfer from prototyping to large scale manufacturing, the compatibility of the cartridges with both diagnostic sectors would significantly save manufacturing costs. At the same time, the biochemistry that offers the species-specificity, i.e., the primers, should be easily exchangeable upon demand, but always keeping the rest of cartridge components the same. 

From the ICT (data management and communication) perspective: Key stakeholders could organize joint workshops so as to bring experts from the two fields under the same roof, making use of ICT platforms and facilities which are increasingly implemented in the majority of the developing countries. Tutorials would add impact to this, especially if they are embedded in platforms that end-users have already been familiar with, such as the Serious Games (Section 4.3). For example, and for a Serious Game addressing entomologists, a mini video could be embedded that would emphasize the importance of sharing data with the human diagnostics community, exchanging information about interventions and results. Thus, it would gradually become a conscience to the malaria program managers that the convergence is of mutual benefit. An issue of utmost importance in ICT implementation is the interoperability between the data management platforms of human diagnostics and vector epidemiology. The impact would be great, as the interoperability between data and diagnostic platforms would lead to a broad database, more efficient surveillance, and early epidemics containment. In addition, interventions would be able to be monitored by individual platforms, for example, an intervention of new insecticide implementation (typical entomological intervention), what influence it has on the reduction (or not) of incidents on humans in the particular region (and perhaps even in combination with specific antimalarial treatment on the patients). Thus, although it is probable that diagnostic tools described in Section 3 may not be used by the same personnel (epidemiologists versus clinicians), the impact is not restricted on a local/health facility/sentinel level, but should be viewed at a higher level: (i) from the entire health system perspective, where data from various sources will be processed altogether, and interventions on mosquitoes and humans will be co-assessed for more informed decisions, management, and control, and (ii) from the manufacturing/procurement perspective, where manufacturers of diagnostics (assays, kits, cartridges, instruments) will find more attractive business models and financial incentives when their market will be unified rather than fragmented. This will, in turn, lead to more manufacturers being active in the field, thus increasing the competition and the quality of the diagnostic and ICT platforms.

From the One Health perspective: an important additional component of a holistic approach is the animal health. Addressing the interplay between malaria, animals, and humans, we make a general distinction between the wildlife and the domestic animals. A striking example of the influence of wildlife in zoonotic malaria is the prevalence in South-East Asia of the *P. knowlesi* species [118]. Its natural host is a macaque monkey, but when it infects humans, it may cause fatal complications. It was only recently, in 2004, that it was recognized to be a cause of zoonotic malaria in humans. Due to morphological similarities with *P. malariae*, the distinction fails under the microscope, thereby causing misinterpretations in the epidemiological mapping, and the necessary interventions. This indicates that molecular assays and diagnostic tools that implement genetic-based identification of the pathogen [119] are needed, as also concluded by the study of Singh et al. [120]. Although the prevalence of *P. knowlesi* is not high in areas of human settlements, but rather, in the wildlife areas where the host animals live, factors like deforestation (or other ecological/environmental causes), human population increase, hunting in the forest, and farming activities, may lead to the *P. knowlesi* changing its host preference from the macaque monkey to human.

The ICT component comes into play with test results on domestic animals: it is known that the *Plasmodium* malaria parasites that infect humans are not infective to livestock (referring to, e.g., sheep, goats, swine, donkeys), therefore, there is no direct risk. However, domestic animals residing in close proximity to people’s households may have an influence on local/district malaria prevalence that is not straightforwardly advantageous or disadvantageous: on one hand, it may be that the vectors are attracted by the animals instead of the humans, therefore, less people are bitten; or inversely, the animals act as additional blood-feeding sources, increase the probability of vectors survival and, being next to the human residences, increase the biting rate on the latter [121]. Results from mathematical models predicting the influence of presence of livestock near people’s residences could be entered as input parameters to the disease data management systems, in order to fine-tune the predictions, and guide more efficient interventions, while providing more complete epidemiological perspectives.

**Figure 4:**
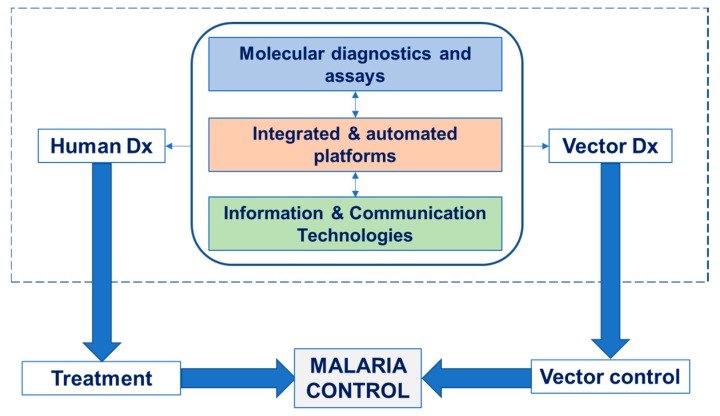
Schematic representation of the interconnection between human and vector diagnostics through the three pathways described in this review (topics within the dashed line). Further related topics of direct relevance, such as treatment (related to vaccines, medicines) and vector control (related to insecticides) are also indicated (outside the dashed line) as components towards the complete One Health approach. Source: K. Mitsakakis, Hahn-Schickard and J. Vontas, FORTH.

Expanding the outreach of this review, even though it has been focused on malaria, it is widely known that other tropical diseases co-exist, especially viral ones that are also vector-borne, for example dengue, chikungunya, West Nile, Zika. The analysis that was conducted in the current review for the convergence between human and vector diagnostics is also valid for the case of the aforementioned vectors: molecular assay development compatibility for detection of the virus in humans and in vectors; novel biochemistries in which the same extraction kit would be able to purify DNA from parasite, and RNA from virus, no matter if it comes from human sample or mosquito homogenate. Even though it may be unlikely that, especially for vector control, tests for malaria and arboviruses would need to be performed simultaneously, still from the economics perspective, it is highly beneficial for the suppliers and manufacturers to have a “one-size-fits-all” platform. Furthermore, it has to be noted that malaria in the context of global health is not only related to diagnosis of humans and mosquitoes (and animals), but also to treatment, as presented in Figure 4. Close interconnection also with environmental features should be taken in consideration during global studies for malaria prevalence, transmission, and elimination [122]. All these create a unifying umbrella, the One Health. Bringing together the human and vector fields can be considered the first step (dashed line in Figure 4, topics discussed in this review). However, there are plenty of other interconnections that could and should be taken into consideration.

## 6. Conclusions

Human diagnostics towards treatment and mosquito diagnostics towards vector control are two aspects of a broad and integrated ecosystem, which, together with animal and environmental health (not analyzed in this review) lead towards a holistic, One Health approach. In this review, we identified three pathways that are common in the fields of human health and mosquito control, namely: biochemical assays, integrated and automated platforms, and information and communication technologies. The authors provided some recommendations, which, through behavioral change from the side of the key stakeholders, could lead to convergence of these pathways and common use by the representatives of the human and vector malaria control programs. Multi-factorial impact is expected: (i) from the intervention perspective, as data from epidemiologists, clinicians, and entomologists will be able to converge and be co-analyzed and co-assessed towards informed decision making; (ii) from the technology perspective, as novel tools and platforms will be developed that are more universal in design and more adaptable in different applications; (iii) from the market perspective, as the converging human and vector diagnostics will generate new business models and more attractive financial incentives for manufacturers and procurers. Starting the convergence for malaria diagnosis and control towards elimination sets the basis for expansion of this combined approach towards other vector-borne diseases, e.g., arboviral diseases.

## Figures and Tables

**Table 1:** Performance characteristics of automated platforms that are already used in malaria diagnosis or are used for other infectious diseases and have the potential to be expanded to malaria.

Technology Platform	Amplification Technology	Portability ^a^	Degree of Multiplexity ^b^	Time-to-Result	Stand-Alone ^c^	Application in Malaria
GeneXpert^®^ (Omni)	RT-qPCR	Benchtop (modular); Omni: portable	Up to 6	60 min	No; Omni: Yes	No
FilmArray^®^	Nested multiplex RT-qPCR; array-based detection	Benchtop	27	60 min	No	No
Alere^TM^ i	NEAR ^d^ (isothermal)	Portable	2	15 min	Yes	No
Enigma^®^ ML	RT-qPCR	Benchtop (modular)	3	95 min	Yes	No
cobas^®^ Liat	RT-qPCR	Portable	3	20 min	Yes	No
EasyNAT^TM^	CPA ^e^ (isothermal); visual readout in lateral flow strip	Handheld	1	90 min	Yes	No
Verigene^®^ RP Flex System	RT-qPCR, gold nanoparticle detection	Benchtop (modular)	16	2 h	Yes	No
GenePOC^TM^	RT-qPCR	Benchtop	12	60 min	Yes	No
Liaison^®^ MDX	RT-qPCR	Benchtop	4	60 min	No	No
VerePLEX^TM^ Biosystem	PCR and microarray hybridization	Benchtop	13	~3.5 h	No	In humans only
Q-POC^TM^	qPCR	Handheld	5	15 min	Yes	In humans only
LabDisk (FeverDisk; demonstrator)	RT-LAMP	Portable	12	2 h	No	In humans
LabDisk (VectorDisk; under development)	RT-qPCR	Portable	36	Target: 2 h	No	In vectors

^a^ “Benchtop” means that it is not transportable. “Modular” means that more than one testing units can be connected to the same, single controller unit; ^b^ number of targets detected per cartridge; ^c^ “Yes” means it does not need a laptop and the handling is integrated on the device; ^d^ nicking enzyme amplification reaction; ^e^ cross priming amplification.
